# Myelodysplastic Syndrome: Clinical Characteristics and Significance of Preclinically Detecting Biallelic Mutations in the *TET2* Gene

**DOI:** 10.3390/life14050637

**Published:** 2024-05-16

**Authors:** Anastasiia Danishevich, Anzhelika Chegodar, Natalia Bodunova, Fedor Konovalov, Maria Nefedova, Natalya Kremneva, Nizhat Kurbanov, Airat Bilyalov, Sergey Nikolaev, Igor Khatkov, Galina Dudina

**Affiliations:** 1 The Loginov Moscow Clinical Scientific Center, 111123 Moscow, Russias.nikolaev@mknc.ru (S.N.);; 2 Independent Clinical Bioinformatics Laboratory, 123181 Moscow, Russia; 3 Institute of Fundamental Medicine and Biology, Kazan Federal University, 420008 Kazan, Russia

**Keywords:** *TET2*, myelodysplastic syndrome, NGS

## Abstract

Myelodysplastic syndrome (MDS) is a clonal disease derived from hematopoietic stem cells, characterized by ineffective hematopoiesis (resulting in peripheral blood cytopenia) and an increased risk of transformation into acute myeloid leukemia. MDS is caused by a complex combination of genetic mutations resulting in a heterogeneous genotype. Genetic studies have identified a set of aberrations that play a central role in the pathogenesis of MDS. In this article, we present a clinical case of MDS transformation into acute myeloid leukemia in the context of two cell lines exhibiting morphological, immunophenotypic, and dysmyelopoiesis markers and the presence of two heterozygous mutations in the *TET2* gene.

## 1. Introduction

Myelodysplastic syndromes (MDS) represent a heterogeneous group of clonal hematopoietic disorders characterized by cytopenia, dysmyelopoiesis, and a high risk of transformation into acute myeloid leukemia (AML) [1]. In Europe and the United States, the incidence rate in the general population is approximately 4–5 cases per 100,000 individuals per year. MDS is rare in children, adolescents, and young adults, with an incidence rate of 0.1 per 100,000 individuals under the age of 40 per year. A notable increase in MDS incidence is observed in individuals aged 70–79, reaching 30.2 cases per 100,000 individuals, and in individuals aged ≥ 80, reaching 59.8 cases per 100,000 individuals [2]. The overall population-based incidence of MDS is approximately 4.9 cases per 100,000 individuals each year [3].

### 1.1. Clinical Characteristics and Diagnosis of MDS

To establish the diagnosis of myelodysplastic syndrome (MDS), bone marrow examination using cytological, histological, cytogenetic, and immunophenotypic methods was conducted. The presence of blast cells during the bone marrow examination, degree of dysplasia, morphological features of hematopoietic dysplasia, degree of stromal fibrosis, and chromosomal abnormalities (Table 1) hold critical significance in the diagnosis of MDS. Fluorescence in situ hybridization (FISH) and flow cytometry should not be considered as the primary standard procedure for assessing the MDS patient’s condition [4].

When evaluating blood smears and bone marrow specimens in MDS, it is essential to consider cytological deviations (Table 2). For the diagnosis of MDS, the recommended number of cells to be examined on the slide was 200 for blood smears and up to 500 for bone marrow [4]. The enumeration of bone marrow blasts holds crucial significance, given its important prognostic value. Berlin blue iron staining (Perl’s stain) should always be performed in MDS cases to assess the presence of ring sideroblasts. The morphological features of dysplasia in MDS, as per the clinical recommendations of ESMO (2020), are summarized in Table 2 [4].

In addition, in 2021, a new classification system for the early stages of myelodysplastic syndrome (MDS) was developed, which included the entities of idiopathic cytopenia of uncertain significance (ICUS) and idiopathic dysplasia of uncertain significance (IDUS). ICUS is characterized by mild cytopenia persisting for at least 4 months (hemoglobin < 11.0 g/dL, neutropenia < 1500/mL, and/or thrombocytopenia < 100,000/mL), the absence of dysplasia or the presence of mild dysplasia (<10%), and bone marrow blasts < 5%. No clonal cytogenetic markers were detected, and other diseases must be ruled out. IDUS, on the other hand, is defined by the absence of pronounced cytopenia (i.e., hemoglobin > 11 g/dL, neutrophils > 1500/mL, and platelets > 100,000/mL), the presence of pronounced dysplasia (>10%), no increase in neutrophilic and/or erythroid and/or megakaryocytic precursors, and bone marrow blasts < 5%. No clonal cytogenetic or molecular markers are present (Table 3) [4,5].

In the absence of distinct morphological features of dysplasia, the diagnosis of MDS can only be established based on the detection of one of the characteristic cytogenetic abnormalities. The diagnosis of MDS cannot be solely based on immunophenotypic analysis using flow cytometry in the absence of characteristic cytological or cytogenetic markers. In some cases, observations with regular repeated examinations of the morphology and karyotype over a minimum period of several months are recommended. Therefore, additional investigations of the bone marrow aspirate, including traditional cytogenetics, flow cytometry, and molecular analysis using Next Generation Sequencing (NGS) methods, are provided in the supplementary information to refine the diagnosis of MDS [6,7].

The additional standardization of cytomorphological features in MDS currently enables the monitoring of patients with minimal manifestations of dysplasia for the timely detection of disease progression and transformation into acute leukemia. The clonal hematopoiesis of indeterminate potential (CHIP) is characterized by the absence of significant cytopenia, absence or mild (<10%) dysplasia, bone marrow blasts < 5%, and the presence of one or more mutations associated with MDS, including, in particular, *DNMT3A*, *ASXL*, *TET2*, *JAK2*, and *TP53* genes, with alternative allele frequencies ranging from 2% to 30%. Clonal cytopenia of undetermined significance (CCUS) is characterized by cytopenia persisting for at least 4 months (hemoglobin < 11.0 g/dL, neutropenia < 1500/μL and/or thrombocytopenia < 100,000/μL), absence or mild (<10%) dysplasia, bone marrow blasts < 5%, and the presence of one or more mutations associated with MDS, including *DNMT3A*, *ASXL*, *TET2*, *JAK2*, and *TP53* genes, with alternative allele frequencies ranging from 2% to 30% [4].

In 2022, the World Health Organization (WHO) reorganized the categories of myelodysplastic syndrome (MDS), focusing on histological and genetic covariates. To decide on the appropriateness of treatment for MDS, it is necessary to stratify patients depending on the degree of acute leukemia (AL) progression risk. To this end, prognostic scoring systems have been developed to stratify patients into low- and high-risk groups based on laboratory characteristics and genetic variant data [8]. Initially, the International Prognostic Scoring System (IPSS) divided patients into four risk groups based on cytopenia, blast percentage, and karyotype. The revised International Prognostic Scoring System IPSS (IPSS-R) divided patients into five groups according to the same parameters, as well as the depth of individual cytopenias. The International Prognostic Scoring System–Molecular (IPSS-M) divided patients into six risk groups using genetic data, providing a more accurate classification. Some patients classified as low risk by the IPSS-R were considered to be at higher risk by the IPSS-M [8].

### 1.2. Molecular Alterations in MDS

When diagnosing MDS, the study of peripheral blood (PB) and bone marrow (BM) is of clinical significance. Chromosomal aberrations can be detected using conventional cytogenetic analysis and FISH, and somatic mutations can be identified using the polymerase chain reaction (PCR), next-generation sequencing (NGS) with targeted gene panels, or exome sequencing [9]. It is known that more than 80% of MDS patients have at least one mutation in genes associated with DNA methylation, chromatin regulation, RNA splicing, transcription regulation, DNA repair, cohesin function, or signal transduction [10]. As the disease progresses and the number of blast cells increases, genetic abnormalities accumulate, which is observed in the majority of newly diagnosed cases of MDS, even in the absence of cytogenetic abnormalities [11]. An increase in the number of driver mutations affects clinical outcomes in patients with MDS [12]. The analysis of MDS tumor samples identified more than 40 target genes, including *SF3B1*, *ASXL1*, *TET2*, *DNMT3A*, *SRSF2*, *RUNX1*, *TP53*, *U2AF1*, *EZH2*, *ZRSR2*, *STAG2*, *CBL*, *NRAS*, *JAK2*, *SETBP1*, *IDH1*, *IDH2*, *ETV6* and others. Some of these mutations have prognostic significance and are associated with the unfavorable clinical course of MDS (Table 4) [2,13].

Mutations in genes involved in the epigenetic regulation of transcription are highly prevalent in patients with MDS. Specifically, recurrent missense, nonsense, splicing sites, and frameshift mutations have been identified in genes associated with DNA methylation, such as *DNMT3A* (DNA methyltransferase de novo) and *TET2* (methylcytosine dioxygenase) [13,14,15]. Loss-of-function mutations in components of histone modification complexes, such as ASXL1, are detected in 10–20% of patients [16,17]. ASXL1 mutations are frequently observed in certain myeloid neoplasms, including MDS, and are associated with poor outcomes [16].

Components of the spliceosome (*SF3B1*, *SRSF2*, *U2AF1*, and *ZRSR2* genes) are mutated in 50–60% of patients with MDS [18,19]. Spliceosome mutations are rarely observed in pediatric myeloid neoplasms, suggesting that they are acquired in elderly individuals [20]. Spliceosome mutations underlie genetic impairments and mutually exclude each other. In fact, the mutant allele burden typically ranges from 40 to 50%, indicating a dominant clone in the bone marrow with a heterozygous mutation [18,19]. In rare cases, MDS with >1 splicing site mutation has been reported, emphasizing the accumulation of these molecular disruptions as a critical factor in the regulation of RNA splicing and their interplay with a single genome [21]. The *SF3B1*, *SRSF2*, and *U2AF1* genes are predominantly characterized by missense mutations at several hotspots, while nonsense and frameshift mutations have not been described [22,23]. Various spliceosome genetic variants are associated with specific clinical phenotypes and varying overall survival/leukemia development risk. Somatic mutations in *SF3B1* are closely associated with MDS patients with ring sideroblasts, with or without thrombocytosis, suggesting a causal relationship between the *SF3B1* mutation and the formation of ring sideroblasts [22,23,24]. Furthermore, the majority of MDS patients with an *SF3B1* mutation exhibit a favorable clinical outcome and a low risk of leukemic transformation [22,23,24]. *SRSF2* mutations are primarily linked to MDS characterized by multilineage dysplasia in the bone marrow and/or blast excess, predicting an unfavorable prognosis and a high risk of leukemia development [22,23]. Somatic *U2AF1* mutations have been described in various MDS subtypes (mostly including forms with multilineage dysplasia and blast excess) and serve as predictors of high leukemia development risk and an unfavorable prognosis with low survival rates [22,23].

Somatic mutations in transcription factors are observed in patients with MDS. The *RUNX1* gene is mutated in 10–15% of patients and is associated with an aggressive disease phenotype, moderate to severe thrombocytopenia, and an unfavorable clinical outcome [22,23,25].

Somatic mutations in the tumor suppressor gene *TP53*, located on chromosome 17p13.1, have been identified in various types of cancer [26]. *TP53* mutations are detected in 5–10% of patients with MDS and are associated with excess blasts and complex karyotypes (including chromosome 17 abnormalities or deletions of chromosomes 5 and 7) [22,23,25]. Patients with MDS harboring *TP53* mutations exhibit an unfavorable clinical prognosis and a high risk of disease progression, and the same holds true for patients with other myeloid neoplasms carrying *TP53* mutations [27].

The oncogenic RAS pathway is the most frequently affected signaling pathway in MDS with recurrent mutations in *NRAS*, *KRAS*, *CBL*, *PTPN11*, and *NF1*. The hyperactivation of RAS signaling is a key driving factor in MDS, characterized by the presence of somatic mutations in *KRAS/NRAS* in 10% of patients [28].

The cohesin complex is a multisubunit protein complex involved in the three-dimensional organization of the human genome and plays a critical role in transcriptional regulation and various DNA repair mechanisms. In 7.5% of patients with MDS, a mutation in the *STAG2* gene, which encodes one of the subunits of the cohesin complex, has been identified. Comparable frequencies of *STAG2* mutations have been reported in patients with AML, suggesting that the altered cohesin function may contribute to the pathogenesis of myeloid leukemia [29].

### 1.3. Clinical Case

A 61-year-old woman sought consultation with a medical geneticist at The Loginov Moscow Clinical Scientific Center in order to assess her risk of developing malignancies and multifactorial diseases.

It is known from the anamnesis that, for the first time in 2014, at the age of 54, during a routine medical examination, a clinical blood test revealed a decrease in leukocytes to the level of 2.44 × 10^9^/L (a decrease in neutrophils to the level of 0.82 × 10^9^/L). The patient was undergoing examination at City Clinical Hospital No. 62 in Moscow. Diagnostic tests did not reveal the presence of a malignant neoplasm. The patient was diagnosed with concomitant diseases, including chronic gastritis, chronic duodenitis, arterial hypertension, and type 2a hyperlipidemia. The family history was burdened with multifactorial diseases and cancer. On the maternal side, the mother, 85 years old, was diagnosed with arterial hypertension, coronary heart disease, cholecystitis, cholangitis, pancreatitis, and Barrett’s esophagus; the grandmother had colorectal cancer and arterial hypertension; and the uncle suffered a myocardial infarction at the age of 50. On the father’s side, the father died in an accident at the age of 70; the grandfather had prostate adenoma; and the patient’s uncle had prostate adenoma at the age of 80. To exclude a high risk of developing malignant neoplasms, as well as the carriage of other clinically significant variants of monogenic diseases, in 2020, the patient was referred for whole-genome sequencing. For 2 years, the woman was regularly observed on an outpatient basis, with periodic general blood tests (Table 5).

As a result of whole-genome sequencing (Table 6), a genetic variant chr2:20141557A>C (c.1922T>G, p.Leu641Ter) in a heterozygous form was identified in exon 18 of the WDR35 gene, which led to the formation of a premature stop codon. In exon 3 of the TET2 gene, the p.Gly908ArgfsTer17 variant, which resulted in a reading frame shift, was identified. In addition, in the 11th exon of the TET2 gene, the p.Gln1903Ter variant, leading to the formation of a premature translation termination site at codon 1903, was identified. Considering the low allelic fraction (~25%) of the detected variants in the TET2 gene, a mosaic form of mutations was hypothesized in both cases.

Based on the patient’s complaints, medical history, and cytological examination of the BM general blood test, morphological examination of peripheral blood, and established platelet anisocytosis (lymphocytes with a rejuvenated nuclear structure—1%—, small lymphocytes with condensed chromatin, their production with a fuzzy nucleolus—58%—, activated lymphocytes—6%—, large granular lymphocytes—35%), a preliminary diagnosis of MDS was established. According to the Revised International Prognostic Scoring System at the time of diagnosis, IPSS-R = 1.00 (very low) and IPSS-M = 3.23 (very low). The hematologist recommended regular monitoring and follow-up with bone marrow cytology every 3 months.

**Figure 1:**
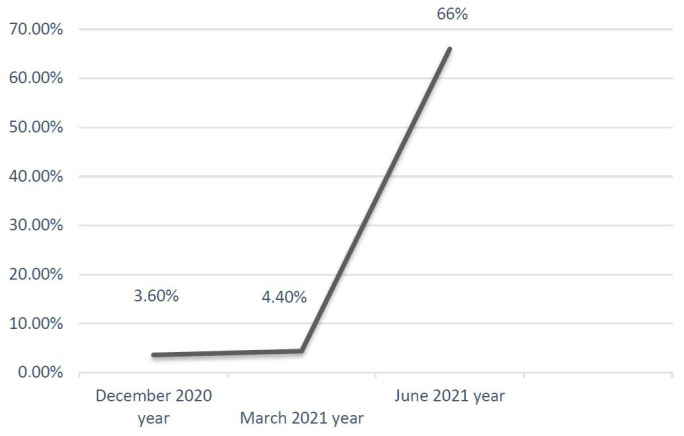
Quantitative changes in BM blast cells over time.

In February 2021, the patient was examined at the National Medical Hematology Center of the Russian Ministry of Health. According to the results of a standard cytogenetic study of the bone marrow, no chromosomal aberrations were detected (46, XX [19]). Unilinear cytopenia (granulocytopenia) was revealed; blasts in the bone marrow amounted to 3.2%. In combination with the presence of morphological abnormalities (granulopoiesis dysplasia in 30–49%, erythropoiesis dysplasia in 10–29%), immunophenotypic markers indicated a high-risk category (combined Oga-ta-Wellscore scale) with dysplastic signs in two cell clones and the presence of two heterozygous mutations in the TET2 gene; therefore, the diagnosis of MDS with multilineage dysplasia was confirmed. The patient’s hematological condition is monitored dynamically, and sternal puncture is performed every three months. In June 2021, a cytological examination of the bone marrow revealed blast cells constituting 66.4% of the total cell mass (Figure 1).

Based on the results of immunophenotyping CD117+CD34-CD33CD13dimcyMPO “++”, the transformation of MDS into AML was observed. The patient underwent chemotherapy treatment. A suitable donor was identified, and on 22 September 2021, allogeneic BM transplantation was performed. Currently, the patient is under the observation of a hematologist.

## 2. Discussion

Protein TET2 is one of the three dioxygenase proteins of the TET (Ten-Eleven Translocation) family, whose primary function is to catalyze the conversion of 5-methylcytosine to 5-hydroxymethylcytosine during DNA demethylation. The *TET2* gene functions as a tumor suppressor, and the haploinsufficiency of this gene initiates myeloid and lymphoid transformations [30]. The disruption of the TET2 protein plays a pleiotropic role in hematopoiesis, including disturbances in stem cell regeneration, clonal determination, and terminal monocyte differentiation. The model of *TET2* mutation impact in hematopoietic stem cells explains the increased regeneration and clonal expansion, leading to the skewing of myeloid clones [31].

When considering a clinical case in a 61-year-old patient, a heterozygous mutation in exon 18 of the *WDR35* gene was identified using whole-genome sequencing; the clinical significance of this variant is described as pathogenic in the Ensemble and dbSNP databases. Mutations of the *WDR35* gene in homozygous or compound heterozygous forms can cause cranioectodermal dysplasia type 2 (Senbrenner syndrome)—a rare heterogeneous ciliopathy with an autosomal recessive pattern of inheritance—which is characterized by the development of skeletal abnormalities, including craniosynostosis, narrow chest, short limbs and brachydactyly, as well as ectodermal defects.

Two heterozygous mutations were identified in exon 3 and codon 11 of the *TET2* gene, respectively. The clinical significance of these variants has not previously been described in databases. However, according to the criteria established by the ACMG, they are characterized as pathogenic clinically significant variants. Considering the low-allelic fraction of the mutant allele and the transformation of MDS into AML, it can be assumed that these mutations are of somatic origin and are in a compound heterozygous state. Thus, these variants potentially contribute to impaired DNA demethylation and potentially play a role in malignant transformation.

According to the literature, mutations in the *TET2* gene are one of the earliest abnormalities observed in MDS and are frequently found in patients with “de novo” MDS. Somatic mutations in the *TET2* gene are rarely observed in individuals younger than 40 years old. However, their frequency increases significantly with age, with clinically significant variants detected in approximately 5–10% of patients over the age of 65 [3]. Xia Wu reported that 39.4% of *TET2* gene mutations in MDS patients may have an inherited nature. These patients manifest MDS at an earlier average age of 48 years, and clinically, a lower percentage of bone marrow blasts is observed [32,33].

However, several scientific groups have sequenced the coding region of *TET2* across a wide range of myeloid malignancies [34,35,36,37,38,39,40,41,42] and have established that mutations in the *TET2* gene are most frequently identified in myeloid malignancies. Various types of genetic variants have been observed, including missense and nonsense, insertions/deletions, and splice site mutations [43]. In our clinical observation, both variants in the *TET2* gene were identified as a frameshift mutation, p.Gly908ArgfsTer17, causing a reading frame shift, and a truncating variant, p.Gln1903Ter, in the heterozygous form.

In many studies, the prognostic factor of *TET2* mutations has been evaluated in patients with MDS, and the results are conflicting [16,44,45,46,47,48,49,50,51,52,53,54]. O. Kosmider et al. reported that mutations in the *TET2* gene were detected in 88 patients with MDS and were identified as an independent favorable prognostic factor in MDS [46]. In another study, Smith et al. reported that *TET2* mutations do not have prognostic significance for patients with MDS and chronic myelomonocytic leukemia [50]. Kim et al. indicated that *TET2* mutations are a poor prognostic factor in patients with MDS [47].

In our clinical case, the patient was under outpatient observation for 2 years, and cytopenia was detected in the CBC. In February 2021, a single lineage cytopenia (granulocytopenia) was identified, with blasts in the bone marrow comprising 3.2%. In conjunction with the presence of morphological findings (dysgranulopoiesis in 30–49% and dyserythropoiesis 10–29%), immunophenotypic features (risk group C according to the Ogata–Well score combined scoring system), dysmyelopoiesis markers in two cell lineages, and the presence of two heterozygous mutations in the *TET2* gene, the diagnosis of MDS with multilineage dysplasia was confirmed. In June 2021, cytological examination of the bone marrow revealed blast cells comprising 66.4%, and based on immunophenotyping results (CD117+CD34-CD33CD13dimcyMPO “++”), the transformation of MDS to AML was confirmed. In this case, it can be hypothesized that genetic variants in exons 3 and 11 of the TET2 gene detected in the patient are poor prognostic factors associated with a high risk of transformation to AML. Further research is required to evaluate the impact of *TET2* gene mutations on the pathogenesis and prognosis of MDS.

Furthermore, there is evidence that patients with *TET2* mutations achieved higher response rates to hypomethylating agents compared to those without mutations, and low *TET2* expression levels in MDS patients were significantly associated with poor overall survival. Thus, the *TET2* expression level may provide additional information for appropriate molecular risk stratification in MDS [55].

## 3. Materials and Methods

Sequencing of a standard whole-genome library prepared from peripheral blood cell DNA was performed using an MGISEQ-2000 sequencer (BGI, Shenzhen, China) with paired reads (2 × 150 bp). A total of 441,269,519 read pairs were obtained, and the effective average genome coverage was 41.2×. After demultiplexing and adapter trimming, sequencing data were aligned to the human genome reference sequence (hg19) using BWA 0.7.12-r1039. Duplicate PCRs were labeled using Samtools 1.12. Freebayes version 0.9.21-18-gc15a283, which was used with default settings to name germline variants. Limitations of the method: The method allows us to identify single-nucleotide substitutions (small insertions and deletions–up to 10 bp) that can cause a genetic disease.

Variant annotation was performed using SnpEff 4.1g based on RefSeq transcripts, including variant population frequencies (1000 genomes, ESP6500, ExAC, gnomAD v.2), pathogenicity prediction scores from the dbNSFP 3.0 database, and information from the dbNSFP 3.0 data. ClinVar. Variant filtering and pathogenicity analysis were performed in accordance with the American College of Medical Genetics and Genomics (ACMG) guidelines. Analysis of allele fractions among individual reads was performed using IGV 2.8.2. The presence of identified variants was confirmed by Sanger sequencing.

## 4. Conclusions

MDS is a clonal hematological disorder characterized by significant genetic and epigenetic complexity and heterogeneity. The diagnosis of MDS involves a wide range of laboratory technologies. However, the clinical case described herein demonstrates the high value of utilizing NGS diagnostic methods for the identification of early molecular-genetic abnormalities leading to MDS, which can be detected as incidental findings during the evaluation of germline mutations.

Mutations identified in the *TET2* gene in individuals with suspected MDS require the development of a personalized monitoring scheme by a hematologist, employing an individualized algorithm of cytological, histological, cytogenetic, and immunophenotypic examinations of the bone marrow. Furthermore, these aberrations may hold promise for risk stratification and informing prognostic and therapeutic decisions for patients with unexplained cytopenia.

## Figures and Tables

**Table 1:** Bone marrow abnormalities in MDS.

Cytological characteristics of bone marrow	Blast cells range from 1% to 19%.
	Dysplasia observed in more than 20% of cells.
Histological examination of bone marrow	Morphological features of hematopoietic dysplasia presented in 1-2-3 hematopoietic cell lineages and the degree of stromal fibrosis expression.
Cytogenetic examination of bone marrow	Chromosomal abnormalities (Routine 20—Metaphase Cytogenetic Analysis).

**Table 2:** Morphological features of dysplasia in MDS.

Specimen Type	Cell Targets	Abnormalities
Peripheral blood	Granulocytes	Pelger–Huët pseudo-cells, abnormal chromatin clumping, hypo-/degranulation, left shift.
	Thrombocytes	Giant platelets and platelet anisocytosis.
	Erythrocytes	Anisocytosis, poikilocytosis, dimorphic erythrocytes, polychromasia, megalocytosis, basophilic stippling, the presence of nucleated erythrocyte precursors, elliptocytes, spherocytes, schistocytes, and fragmentocytes.
Bone marrow	Bone marrow cellularity	Typically hypercellular, with rarely decreased cell count.
	Erythrocytes	Macrocytes, multinucleated erythrocytes, protrusions, non-round nuclei, karyorrhexis, nuclear bridges, atypical mitoses, sideroblasts, ring sideroblasts, erythroid precursors, and those detected during Shik resection.
	Megakaryopoiesis	Micromegakaryocytes, mononuclear megakaryocytes, dumbbell-shaped nuclei, hypersegmentation, and multiple nuclei.
	Granulopoiesis	Left shift, increased blast content in the bone marrow, Auer rods, hypo/degranulation, Pelger–Huët pseudo-cells, nuclear anomalies (e.g., hypersegmentation, abnormal chromatin condensation), myeloperoxidase deficiency, and monocytosis with morphological alterations of monocytes.

**Table 3:** Classification of early stages of MDS.

Condition	Clinical Manifestations
Idiopathic cytopenia of undetermined significance	Mild cytopenia persisting for 4 months (hemoglobin < 11.0 g/dL, neutropenia < 1500/mL, and/or thrombocytopenia < 100,000/mL).
	No dysplasia or mild dysplasia (<10%) is observed.
	Blasts in the bone marrow < 5%.
	No clonal cytogenetic markers are present.
	Rule out other diseases.
Idiopathic dysplasia of undetermined significance	Absence of pronounced cytopenia (i.e., hemoglobin > 11 g/dL, neutrophils > 1500/mL, and platelets > 100,000/mL).
	Severe dysplasia (>10%) of the neutrophilic and/or erythroid and/or megakaryocytic lineage.
	Blasts in the bone marrow < 5%.
	There are no clonal cytogenetic or molecular markers present.

**Table 4:** Most common somatic mutations observed in MDS.

Gene Function	Gene	Frequency (%) *
Epigenetic regulators and chromatin remodeling factors	*TET2*	15–25
	*ASXL1*	10–20
	*DNMT3A*	10
	*IDH1/2*	5–10
Pre-mRNA splicing factors	*SF3B1*	15–30
	*SRSF2*	10–15
	*U2AF1*	5–10
Transcription factors	*RUNX1*	10–15
	*TP53*	5–10
Signaling molecules	*NRAS/KRAS*	10
Cohesin complex	*STAG2*	5–7

* Other mutations are observed in <5% of cases.

**Table 5:** Dynamics of CBC parameters.

Parameters CBC	Date (Year)									
	2014		2015	2016	2017	2018	2020		2021	
	08.02	08.10	30.01	21.06	03.06	21.08	30.09	26.12	22.05	08.06
PLT (109/L)	246	236	172	204	190	188	177	202	194	160
WBC (109/L)	4.56	3.92	3.9	3.07	3.28	3.76	2.44	3.38	2.23	1.64
NE (%)	42	40	55	39.4	37.9	32.7	33.5	-	-	-
NE# (109/L)	1.92	1.57	2.3	1.21	1.24	1.23	0.82	0.88	0.45	0.3
NEBF (%)	3	2	4	<6	-	1	3	1	1	1
NESN (%)	39	38	48	-	-	31	38	25	19	17
MON (%)	15	9	9.5	13.4	10.5	12.49	2.6	15	12	6
MON# (109/L)	0.68	0.35	0.3	0.41	0.34	0.47	0.31	0.51	0.27	0.1
LYM (%)	36	46	35	42	48.5	53.07	44.9	59	68	76
LYM# (109/L)	1.64	1.8	1.3	1.29	1.59	2.0	1.09	1.99	1.52	1.25
EO (%)	7	5	1	4.9	2.5	1.37	5.6	0	0	0
EO# (109/L)	0.32	0.2	-	0.15	0.08	0.05	0.14	0	0	0

PLT—platelet count; WBC—white blood cells; NE—neutrophils; NEBF—band forms neutrophils; NESN—segmented neutrophils; MON—monocytes; LYM—lymphocytes; and EO—eosinophils.

**Table 6:** Whole-genome sequencing of germline DNA.

Gene	HGVS Names (hg19)	Zygosity (Allelic Fraction of Minor Allele)	MAF (1000 Genomes, ESP6500, ExAC, gnomAD)	Clinical Significance (ACMG)
*WDR35*	chr2:20141557A>C	heterozygous	gnomAD (0.0204)	Pathogenic
	c.1922T>G			
	p.Leu641Ter			
	ENSG00000118965			
*TET2*	chr4:106157821insAGAC	Potentially somatic (~25%)	Not found	Likely Pathogenic
	c.2721_2722insAGAC			
	p.Gly908ArgfsTer17			
	ENST00000380013.9			
*TET2*	chr4:106197374C>T	Potentially somatic (~25%)	Not found	Likely Pathogenic
	c.5707C>T			
	p.Gln1903Ter			
	ENST00000380013.9			

## Data Availability

The data are not publicly available due to restrictions as they contain information that could compromise the privacy of research participants. Requests to access the additional data should be addressed to the following email: s.nikolaev@mknc.ru.
